# The predictive role of biochemical markers on outcomes of severe COVID-19 patients admitted to intensive care unit

**DOI:** 10.5937/jomb0-40641

**Published:** 2023-08-25

**Authors:** Bosa Mirjanić-Azarić, Ivana Pejić, Smiljana Mijić, Aleksandra Pejčić, Anita Đurđević-Svraka, Dragan Svraka, Darija Knežević, Tatjana Milivojac, Nataša Bogavac-Stanojević

**Affiliations:** 1 University of Banja Luka, Medical Faculty, Department of Medical Biochemistry, Banja Luka, Republic of Srpska, Bosnia and Herzegovina; 2 University Clinical Centre of the Republic of Srpska, Banja Luka, Republic of Srpska, Bosnia and Herzegovina; 3 General Hospital Gradiska, Gradiska, Republic of Srpska, Bosnia and Herzegovina; 4 Aqualab Laboratory, Banja Luka, Republic of Srpska, Bosnia and Herzegovina; 5 University of Banja Luka, Medical Faculty, Banja Luka, Republic of Srpska, Bosnia and Herzegovina; 6 University of Belgrade, Faculty of Pharmacy, Department of Medical Biochemistry, Belgrade

**Keywords:** COVID-19, neutrophil-to-lymphocyte ratio, systemic inflammation index, D-dimer, magnesium, ferritin, COVID-19, odnos neutrofili-limfociti, sistemski inflamatorni indeks, D-dimer, magnezijum, feritin

## Abstract

**Background:**

The pandemic of severe acute respiratory syndrome by coronavirus 2 (SARS-CoV-2) is a multi-system disease caused by a diffuse systemic process involving a complex interaction of the inflammatory, immunological and coagulative cascades. This study aims to identify the most effective biomarkers to predict poor outcome in intensive care unit (ICU) patients with severe COVID-19 disease.

**Methods:**

A single-centre retrospective observational study enrolled 69 deceased and 20 recovered patients treated in the ICU of the General Hospital Gradiska in the period from March 1, 2021. until April 1, 2022. We evaluated the leukocytes (WBC), lymphocytes (LYM), neutrophils (NEU), platelets (PLT), haemoglobin, neutrophil-lymphocyte ratio (NLR), platelet lymphocyte ratio (PLR), and systemic immune-inflammation index (SII). In addition, we evaluated the IL-6, ferritin, CRP, D-dimer, magnesium, bilirubin and lactate dehydrogenase.

## Abstract (sr)

**Uvod:**

Pandemija teškog akutnog respiratornog sindroma koronavirusa 2 (SARS-CoV-2) je multisistemska bolest izazvana difuznim sistemskim procesom koji uključuje kompleksne interakcije inflamatornih, imunoloških i koagulacionih kaskadnih procesa. Cilj ove studije je da utvrdi najefikasnije biomarkere u proceni lošeg ishoda pacijenata u jedinici intenzivne nege (ICU) obolelih od teškog oblika bolesti COVID-19.

**Metode:**

Retrospektivna opservaciona studija je uključivala 69 preminulih i 20 preživelih pacijenata lečenih u ICU u Opštoj bolnici Gradiška u periodu od 01.03.2021. do 01.04.2022. godine. Procenjivane su vrednosti leukocita (WBC), limfocita (LYM), neutrofila (NEU), trombocita (PLT), hemoglobina, odnosa neutrofila i limfocita (NLR), odnosa trom bocita i limfocita (PLR), sistemskog inflama tornog indeksa (SII). Takođe, procenjivane su vrednosti inter leukina6, feritina, CRP, D-dimera, magnezijuma, bilirubina i aktivnost laktat dehidrogenaze.

**Rezultati:**

Prilikom prijema pacijenata u ICU postojala je značajna razlika u vrednostima sledećih parametara između preminulih i preživelih pacijenata: WBC x 109/L 11,50 (8,86-14,75) prema (vs.) 9,40 (5,90-11,90), p=0,026; NEU x109/L 10,15 (7,81-12,74) vs. 8,60 (4,80-10,30) p=0,022; NLR 15,01 (10,60-24,33) vs. 9,45 (5,10-14,90) p=0,02; SII 3712 (2240-6543) vs. 1949 (993-3720) p=0,003. Takođe, tokom vremena koncentracija magnezijuma je značajno rasla u grupi preminulih pacijenata, dok je koncentracija hemoglobina i broj trombocita opadao. ROC analiza je pokazala sledeće AUC vrednosti: WBC 0,774; NEU 0,781; NLR 0,786; SII 0,776; D-dimer 0,741 i bilirubin 0,713.

**Zaključak:**

U ovoj retrospektivnoj studiji WBC, NEU, NLR, SII, D-dimer i bilirubin, određeni pri prijemu u ICU, su imali visoku vrednost u predviđanju smrti između pacijenata s teškim COVID-19.

## Introduction

In December 2019, a novel coronavirus (SARS-CoV-2) caused a severe coronavirus disease(COVID-19) pandemic. Most infected people developed a mild to moderate illness and recovered without hospitalization. However, some became seriously ill with a systemic inflammatory response leading to acute respiratory distress syndrome and cardiovascular events demanding intensive therapy. COVID-19 in critically ill patients is represented as multi-system disease caused by a diffuse systemic process involving a complex interaction of the inflammatory, immunological and coagulation cascades [1].

Biochemical markers are potential predictors for the prognosis of clinical outcomes for COVID-19 patients, especially those that represent inflammation, coagulation and immune status. A peripheral leukocytes (WBC) count, neutrophil-to-lymphocyte ratio (NLR), platelet-to-lymphocyte ratio (PLR) and systemic immune-inflammation index (SII) are indicators of the systematic inflammatory response [2] [3]. The studies showed that SII, as a relatively new inflammatory index based on peripheral lymphocytes (LYM), neutrophils (NEU), and platelets (PLT) counts could have helped in predicting the clinical outcome of patients with COVID-19 [4]. Interleukin-6 (IL-6), inflammatory cytokine released by macrophages increase in patients with severe COVID-19 disease and is associated with complications in COVID-19 patients with the development of cytokine storm, leading to damaged lungs and other organs [5] [6]. Also, measurements of plasma inflammatory markers such as ferritin, C-reactive protein (CRP) and procalcitonin (PCT) could be used to predict COVID-19 progression. Until recently, ferritin was rarely used as a marker of inflammation, but numerous recent studies of COVID-19 have shown that ferritin plays a significant role in the prognosis of patients with COVID-19 [7] [8]. Ferritin has a defensive role in the human organism by limiting the supply of iron to the pathogen, and in addition regulates the synthesis and release of cytokines responsible for the cytokine (pro-inflammatory) storm [9]. Well-known early markers ofinflammation or infection CRP may be useful for early detection of severity during COVID-19 [10]. Also, PCT has been identified as a predictors of disease severity in COVID-19 and it was previously used for distinguishing viral infections from bacterial infections [11] [12] where elevated PCT levels were often found in patients with bacterial infections.

Regarding blood coagulation as a risk factor for death, recent literature data show that D-dimer values are higher in patients with severe COVID-19 than in those with milder forms and could be used to predict COVID-19 progression [13]. Thrombocytopenia is a common laboratory abnormality found in severely ill patients, and is associated with poor clinical outcomes, including death. Likewise, lower PLT counts in patients hospitalized with COVID-19 are associated with poorer clinical outcomes [14].

This study aims to evaluate and identify the most effective biomarkers that could be used in clinical practice to predict the poor outcomes in ICU patients with COVID-19. The identification of predictive biomarkers would mean a better risk stratification of the patients and thus offers a greater possibility for successful treatment of patients with COVID-19.

## Materials and methods

### Subjects

A single-center retrospective observational study enrolled all consecutive patients with confirmed SARS-CoV-2 infection was conducted of 115 patients (n=115) treated in the ICU of the General Hospital Gradiska in the period from March 1, 2021. until April 1, 2022. After the ethical committee approval, the data were excluded from the patient’s medical histories. Patients treated uniformly according to the national protocol issued by the Republic of Srpska Ministry of Health for the treatment of COVID-19 disease caused by the SARS-CoV 2 virus were processed.

Factors for the inclusion of patients in the study included: proven SARS-CoV-2 infection by the RT-PCR method, acute respiratory distress syndrome that required treatment in the ICU, i.e. some type of mechanical ventilation, length of hospitalization in the intensive care unit for more than 5 days. The final number of included patients was 89. The 26 patients at the terminal stage of malignant disease, the terminal stage of cardiovascular disease, and patients in the ICU for less than 5 days were excluded from the study.

The study includes 69 deceased and 20 recovered patients. The disease outcome was followed along with the hospital course of every patient at the time of analysis. The medical history, demographic characteristics, comorbidities, disease severity at admission, and laboratory findings were collected. The final outcome of these admitted patients was categorized as deceased or recovered. Laboratory results were collected at baseline (at the time of ICU admission) and at three-time points before the patient’s final outcome. Their change compared to the initial values was also recorded.

Likewise, the criteria for treating patients with tocilizumab (TCZ) were: IL-6 >40 pg/mL and CRP >100 mg/L at the first point of the following (base values).

### Laboratory parameters

We measured the following blood cell count inflammation parameters: WBC count, LYM, NEU, PLT, red blood cells and haemoglobin. Then we assessed combined blood cell indexes of systemic inflammation: NLR = absolute NEU count/absolute LYM count; PLR = absolute PLT /absolute LYM; SII= thrombocyte count × NEU count/LYM count.

We also measured the following biochemical markers: IL-6, ferritin, CRP, PCT, fibrinogen, D-dimer,lactate, calcium, magnesium, albumin, total protein, bilirubin, creatinine, urea, activated partial thromboplastin time (aPTT); partial pressure of carbon dioxide (pCO2); aspartate aminotransferase (AST); alanine aminotransferase (AST); lactate dehydrogenase (LDH). All tests were performed according to the product manual.

### Statistical analysis

Data distribution was tested using the Kolmogorov-Smirnov test. Normally distributed independent data were compared by Student’s t-test. Skewed data were compared by the Mann-Whitney U test. Categorical variables were tested by the Chi-square test. Two-way repeated measured ANOVA and Fridman signed-rank test was used to test whether the changes in four repeated observations were statistically significant. Data are shown as mean ± standard deviation for normally distributed variables. The median for independent data or the median of difference for dependent data with interquartile range were presented for non-normally distributed variables. The accuracy of laboratory parameters for discrimination between deceased and recovered patients was calculated by applying the receiver operating curve (ROC) analysis. Optimal cut-off values were calculated only for laboratory parameters with useful clinical accuracy. Relative or absolute frequencies are shown for categorical variables. Kaplan-Meier survival analysis and the log-rank test were used to assess and compare changes in lethal outcome frequencies over time in patients divided into two groups according to cutoff values from the ROC analyses. A univariate and multivariate Cox regression analysis was applied to the observed time-to-outcomes survival data to estimate hazard ratios (HR) and adjust relevant confounders. The date was presented as HR with a 95% confidence interval (CI). Analyses were conducted by the statistical programme SPSS 27.0 (SPSS Inc. Chicago, USA). A two-tailed p-value 0.05 was considered to indicate statistical significance.

## Results

Table 1 presents demographic and clinical data of 89 SARS-CoV-2 infected patients classified according to survived and lethal outcomes. No difference between genders was observed, but the deceased patients were older than the recovered ones. From comorbid conditions before SARS-CoV-2 infection, only immunological disorders were prevalent in the recovered group. Significantly more recovered patients were on high-flow oxygen therapy. However, 90% of decreased patients received mechanical ventilation.

**Table 1:** Demographic and clinical data of 89 SARS-CoV-2 infected deceased and recovered patients.

Parameters	Deceased<br>patients<br>n (69)	Recovered<br>patients<br>n (20)	p
Age, years	70±8.2	63±12.4	0.002
Gender-male, %	65.2	60.0	0.669
Oxygen<br>supplementation, n (%)	2 (2.9)	11 (55)	0.001
Intubation, n (%)	62 (89.9)	4 (20)	0.001
Cardiovascular disease,<br>n (%)	53 (76.8)	13 (65)	0.288
Cardiomyopathy, n (%)	14 (20.3)	3 (15)	0.596
Myocardial infarction,<br>n (%)	2 (2.9)	1 (5.3)	0.647
Diabetes mellitus, n (%)	15 (21.7)	6 (30)	0.444
Cancer, n (%)	2 (2.9)	1 (5.3)	0.539
Immunological disorders,<br>n (%)	1 (1.4)	3 (15)	0.034
Hypertension, n (%)	49 (74.2)	11 (55)	0.168
Thyroid disease, n (%)	2 (2.9)	11 (55)	0.523

Table 2 shows results for hematological, inflammatory and biochemical parameters in examinedpatient groups. WBC and NEU counts were significantly lower in recovered patients along withcalculated inflammatory indexes (NLR, PLR and SII). Lower creatinine values were also recorded in recovered patients.

**Table 2:** Laboratory parameters in the first point in deceased and recovered patients. Data are presented as mean value ± standard deviation and compared by the Student t-test. ^*^Data are presented as median (interquartile range) and compared by the Mann-Whitney U test. Neutrophile-to-lymphocyte ratio (NLR); platelet-to-lymphocyte ratio (PLR); systemic immune-inflammation index (SII); C- reactive protein (CRP); activated partial thromboplastin time (aPTT); partial pressure of carbon dioxide (pCO_2_); aspartate aminotransferase (AST); alanine aminotransferase (AST); lactate dehydrogenase (LDH); reference interval (RI).

Parameters, units, RI	Deceased patients<br>(n = 69)	Recovered patients<br>(n = 20)	p
Haematological parameters			
Leukocytes x10^9^/L (3.40–9.70)	11.50 (8.86–14.75)	9.40 (5.90–11.90)	0.026^*^
Neutrophiles x10^9^/L (2.06–6.49)	10.15 (7.81–12.74)	8.60 (4.80–10.30)	0.022^*^
Lymphocytes x10^9^/L (1.19–3.35)	0.64 (0.46–0.93)	0.90 (0.51–1.13)	0.131^*^
Platelets x10^9^/L (158.00–424.00)	244.15±81.10	244.80±95.43	0.976
Haemoglobin, g/L (m:138.00–175.00;<br>f:119.00–157.00)	132.26±17.97	135.80±20.03	0.452
Inflammatory parameters			
NLR	15.01 (10.60–24.33)	9.45 (5.10– 14.90)	0.002^*^
PLR	351.43 (250.30–549.20)	257.50 (189.90–383.40)	0.074^*^
SII	3712 (2240–6543)	1949 (993–3720)	0.003^*^
IL-6, pg/mL (< 7.0)	7 1 . 0 ( 3 0.5–108.2)	36.5 (13.5–175.0)	0.122^*^
Ferritin, ng/mL (11–306)	964 (577–1360)	667 (350–1257)	0.428^*^
CRP, mg/L (<5.0)	103.0 (47.0–157.0)	125.0 (67.0–137.0)	0.598^*^
Procalcitonin, ng/mL (<0.050)<br>(0.05–0.5 local infections)<br>(0.5–2.0 sepsis; >2.0 severe sepsis)	0.195 (0.090–0.538)	0.185 (0.075–0.655)	0.910^*^
Fibrinogen, g/L (1.80–3.50)	3.80 (3.00–4.85)	4.50 (3.25–6.45)	0.119^*^
Parameters of the hemostatic system			
D-dimer, mg/L (<0.50)	4.64 (1.54–16.75)	2.17 (0.92–4.13)	0.091^*^
APTT, sec (22.00–35.00)	35.24±33.50	32.81±32.20	0.307
Parameters of aerobic capacity			
Lactate, mmol/L (0.500–2.200)	1.947±1.299	1.541±0.536	0.212
pCO_2_, mmHg (35.000–45.000)	34.493±7.167	35.263±8.339	0.690
Biochemical parameters and enzymes			
Calcium, mmol/L (2.14–2.53)	2.10±0.14	2.11±0.20	0.702
Magnesium, mmol/L (0.65–1.05)	0.86±0.22	0.87±0.13	0.951
Albumin, g/L (34.00–55.00)	30.25±2.09	31.35±3.69	0.169
Total protein, /L (64.00–83.00)	62.71±6.56	62.05±5.22	0.690
Creatinine, μmol/L (m:62.0–106.0;<br>f:44.0–80.0)	85.0 (76.0–103.0)	77.5 (68.0–93.0)	0.049^*^
Urea, mmol/L (3.09–9.20)	10.65±6.30	8.32±4.29	0.124^*^
Bilirubin, μmol/L (1.70–21.00)	14.36±9.63	10.00±4.52	0.053
AST, IU/L (5.0–34.0)	62.0 (45.0–104.5)	68.5 (36.0–83.5)	0.630^*^
ALT, IU/L (5.0–50.0)	51.0 (29.5–105.0)	52.0 (34.5–82.0)	0.810^*^
LDH, IU/L (125.0–250.0)	653.5 (483.5–801.5)	597.5 (443.0–846.5)	0.608^*^

**Figure 1:**
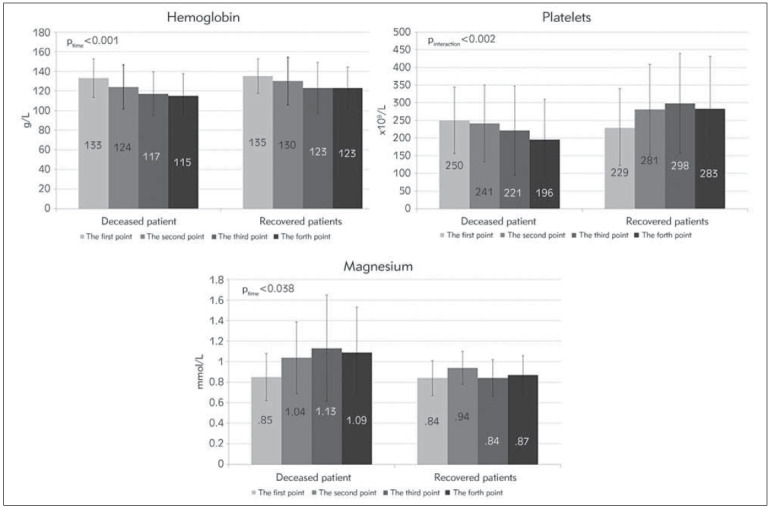
Significantly changed normally distributed parameters over time. (Mean values ±standard deviation).

We further analyzed changes in examined parameters over time. The results demonstrated thatmag nesium concentrations significantly increased over time in the group of deceased patients. At the same time, we observed a reduction in the haemoglobin concentration in deceased patients. Asignificant interaction between lethal and survived outcomes and time was demonstrated for the platelets. The platelet count was reduced in deceased patients but increased in those who recovered (Figure 1).

**Figure 2:**
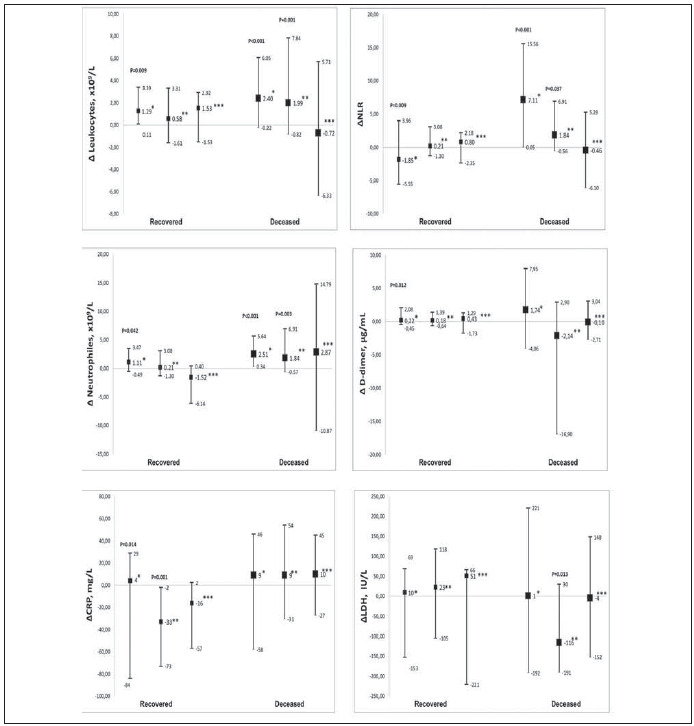
Significantly changed skewed parameters over time. (Median of difference and 25. and 75. quartile). ^*^ changes between II and I point; ^**^ changes between III and II point; ^***^ changes between IV and III point

The median of difference between the four-time points revealed that the WBC and NEU count significantly increased over time in both groups. The rise in the WBC and NEU count was observed in the second point in recovered patients and the second and third points in deceased patients. A significant reduction of LDH activity was demonstrated only in deceased patients with a concomitant increase of NLR. The NLR index significantly changed over three-time points, in contrast to LDH activity that changed between the first two points. In recovered patients, only CRP concentrations decreased, and D-dimer increased over time. The median of difference between the second and first points indicates a slight rise in CRP concentration. In almost 50% of patients, a reduction in concentration was recorded. A significant decrease in CRP concentration in about 75% of patients was shown between the third and second-time points. However, a slight increase in D-dimer concentrations was observed only for the second point (Figure 2).

In the case of ferritin concentration, significant changes over time were not shown but deceased patients had significantly higher concentrations in the third 1259 (660–1500) ng/mL and in the fourth point 1500 (1257–1500) ng/mL compared to recovered ones [540 (337–739) ng/mL, p=0.009 for the third and 472 (347–890) ng/mL, p=0.029 for the fourth point].

In the future analysis, we investigated the accuracy of examined parameters in the first point for discrimination between deceased and recovered patients. Table 3 presented only significant AUCs with useful discrimination ability. Therefore, only WBC, NEU, NLR, D-dimer, SII and bilirubin had thepotential to be useful diagnostic biomarkers. Additionally, we analyzed the optimal cut-off values calculated by the ROC analysis. WBC and D-dimer had better specificity than sensitivity, opposite to NEU, NLR and bilirubin, whereby sensitivity was higher than specificity. SII had a sensitivity value similar to specificity.

**Table 3:** ROC analyses for the screening of deceased patients with useful accuracy Neutrophile-to-lymphocyte ratio (NLR); systemic immune-inflammation index (SII), area under the curve (AUC), confidence interval (CI).

Parameter	AUC<br>(95%CI)	Optimal<br>cut-off	Sensitivity	Specificity	p
Leukocytes, x10^9^L	0.774 (0.684–0.864)	10.4	0.643	0.810	<0.001
Neutrophiles, x10^9^L	0.781 (0.687–0.876)	6.3	0.886	0.568	<0.001
NLR	0.786 (0.680–0.873)	8.0	0.916	0.568	<0.001
D-dimer	0.741 (0.651–0.831)	2.4	0.643	0.3787	<0.001
SII	0.776 (0.680–0.873)	2387	0.714	0.730	<0.001
Bilirubin	0.713 (0.587–0.838)	9.5	0.714	0.682	<0.001

**Figure 3:**
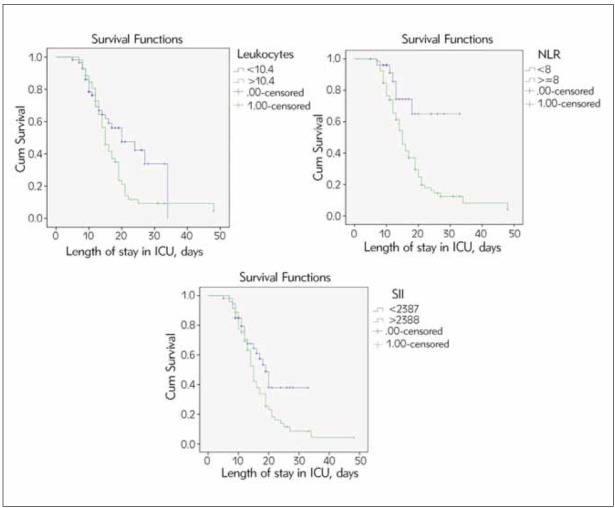
Kaplan–Meier curves of leukocytes, D-dimer, NLR and SII for the lethal outcome.

We used cut-off values from ROC analysis to split patients into two groups (with values above andbelow the cut-off) to perform Kaplan-Meier survival analysis to estimate the association with lethal outcomes. A statistically significant association was observed between WBC, D-dimer, NLR and SII categories and the lethal outcome (Figure 3). In patients with WBC, D-dimer, NLR, and SII above cut-off values, lethal outcomes were seen in 85%, 81.5%, 80% and 83% cases, respectively. However, the lethal outcome was seen in 42%, 41%, 22% and 43% of patients with values below the cut-off for WBC, D-dimer, NLR and SII, respectively. Cox regression analysis was employed to analyze the association between the high values of previously mentioned parameters measured in the first time point and the risk of fatal outcomes. The results showed that values above the cut-off for WBC, D-dimer, NLR and SII were associated with increased mortality (Table 4). After the inclusion of all significant predictors in multivariate analyses, the independent predictor of lethal outcomes was NLR values above 8. Patients with values above 8 had a 3.4 times higher risk for lethal outcomes than those below 8.

**Table 4:** Hazard ratios for lethal outcomes in univariate and multivariate COX regression. Neutrophile-to-lymphocyte ratio (NLR); systemic immuneinflammation index (SII), Hazard ratio (HR), confidence interval (CI).

Parameter	HR (95%CI)	p
Leukocytes, x10^9^L	1.753 (1.071–2.869)	0.026
Neutrophiles, x10^9^L	1.503 (0.866–2.610)	0.147
NLR	3.173 (1.371–7.342)	0.007
D-dimer	1.760 (1.079–2.873)	0.024
SII	1.725 (1.016–2.929)	0.043
Bilirubin	1.631 (0.967–2.751)	0.067
All predictors in themodel SII<br>NLR<br>D-dimer	1.014 (0.552–1.861)<br>3.442 (1.238–9.570)<br>1.355 (0.797–2.303)	0.964<br>0.018<br>0.261

The results showed that D-dimer values were in the groups with TCZ vs. without TCZ: recoveredpatients 1.63 (0.88–2.26) vs. 6.70 (3.2820.63), p=0.007 and deceased patients 1.70 (0.63–12.9) vs. 4.36 (1.20–16.40), p=0.468. In addition, there was no difference in D-dimer values in the recovered vs. deceased patients with TCZ: 1.63 (0.88-2.26) vs. 1.70 (0.63–12.9).

## Discussion

We conducted a retrospective analysis of 89 COVID-19 patients in the hospital ICU. We found that inflammatory marker WBC, NEU, NLR and SII were significantly raised in non-survivors compared to survivors in ICU on admission. Numerous studies were conducted to determine the relationship between these hematologic markers and the overall outcome of patients with COVID-19 disease. NEU, the most numerous circulating WBCs, are a significant immune system component. They are the first line of innate immune defense and have a protective role during bacterial and fungal infections, killing pathogens by phagocytosis. In viral infections, it is assumed that NEU activate the immune system and release reactive oxygen species that can cause damage to cellular DNA and remove the virus from the cells, which is later targeted by antibodies [15]. In addition, NEU trigger the production of various cytokines and effector molecules [15]. However, their role in viral infections remains unclear. A study conducted on mice infected with SARS-CoV-2 showed that NEU were not crucial in the removing of the virus from the lung cells and the host’s survival [16].

The role of LYM in viral infections is much clearer. Viral infection causes a predominantly LYMresponse, but systemic inflammation, especially high levels of IL-6, paradoxically reduces LYM numbers and consequent cellular immunity [15]. Also, the lymphopenia observed in COVID-19 patients was related to the ability of the virus to infect T cells through the angiotensin-converting enzyme 2 (ACE2) receptor and CD147-spike protein [16]. NLR is the most studied biomarker in COVID-19 and represents an independent risk factor for in-hospital mortality in these patients, especially men [17]. Several studies have confirmed its predictive value for severe disease progression and mortality [17] [18]. Data from our group showed that NLR predicted in-hospital mortality in COVID-19 patients with an accuracy of 0.786 (0.68-0.83) and sensitivity and specificity of 91.6% and 56.8%, respectively if NLR cut-off value is equal to 8.

The results of our study are further confirmed with current knowledge about NLR, from two largemeta-analyses that found that patients with severe symptoms and non-survivors of COVID-19 had higher levels of NLR on admission than non-severe patients and survivors [19] [20]. In addition, our study showed that another index of inflammation derived from the number of blood cells, such as SII, was significantly increased in the deceased, compared to surviving COVID-19 patients. These results concur with previous studies on its diagnostic role in SARS-CoV2-infected patients [4] [21]. SII includes three parameters of peripheral blood: the number of NEU, PLT and LYM, which represent the balance of the immune and inflammatory status of the host. Even though it is a newer marker, it has been proposed that SII represents a prognostic biomarker in patients with sepsis [22]. Recently, a retrospective study by Fois et al. showed that SII is the most significant prognostic biomarker for the survival of patients with SARS-CoV2 [4].

D-dimer is a fibrin degradation product and its main utility is in the diagnosis and management of thrombotic disorders. This study found that D-dimer values on hospital admission were 2 times higher in none survived compared to survived patients (4.64 vs. 2.17 mg/L, respectively). Elevated D-dimer and thrombotic complications have been widely reported in COVID-19 patients. The study in China found that an admission D-dimer value greater than 2 mg/L was associated with increased probability of mortality (odds ratio 10.17 (95% CI 1.10–94.38) [23]. These results comply with other studies indicating the enormous importance of this parameter in predicting fatal outcomes in COVID-19 patients [24]. A survey by Soni et al. [25] found that the optimal D-dimer cutoff value for the prediction of in-hospital mortality was 1.44 mg/L. In contrast, Yao et al. [23] found that the optimal cut-off value in hospitalized patients for the same outcome was 2.01 mg/L. In compliance with the before-mentioned, our study found that D-dimer had the predictive capability for a lethal outcome, with an AUC equal to 0.741. The most common causes of D-dimer elevation are viremia and cytokine storm syndrome, in which an increase in pro-inflammatory cytokines cannot be controlled by early anti-inflammatory factors [24]. In our study, we observed that patients treated with TCZ had reduced D-dimer levels at four follow-up points, indicating that TCZ could reduce the risk of thrombotic events. These results are in accordance with the study by Hassan at al. [26]. Gualtierotti et al. [27] reported that TCZ decreases factor XIII (FXIII) plasma levels. FXIII is involved in the coagulation cascade, and its deficiency is associated with a bleeding syndrome, while FXIII excess is related to thrombosis. This mechanism could explain why patients on TCZ treatment had significantly lower D-dimer values.

Although there was no notable difference in PLT in patients on admission to the ICU, we noted a significant change in their PLT number during treatment. The number of PLT increased in survivors and decreased in non-survivors.

Thrombocytopenia is frequent in ICU patients, leading to blood coagulation disturbances and increasing the risk of death. The possible mechanism of thrombocytopenia is different, as stated in the study by Jiang et al. [28]. However, why the PLT count decreases in COVID-19 patients with a poor outcome is not clear and needs further investigation. Inflammatory markers, including IL-6, ferritin, CRP and PCT, show a prognostic significance in patients with COVID-19 disease. The cause of patient deaths with severe COVID-19 is considered to be the cytokine storm that occurs in infections and leads to the release of pro-inflammatory factors [19].

Although no statistically significant difference was found in the levels of IL-6 and ferritin between non-survivors and survivors, the IL-6 and ferritin mean concentrations were 2 and 1.5 times higher,respectively, in the non-survivor group. In our study, we observed that the values of IL-6 were 10 times higher at the last follow-up point in non-survivors (results not shown).

Ferritin was associated with poor prognosis and could predict a deterioration in the condition of COVID-19 patients [29]. In the present study, the last point is followed by the ferritin level in non-survivors >1500 ng/mL, which is 3–4 times higher than in survivors. On admission, ferritin concentration cannot reliably predict a poor outcome. Still, but we believe that longitudinal monitoring of ferritin during hospitalization can help predict poor progression in patients with COVID-19. Cheng et al. investigated the association of ferritin levels with poor outcomes in COVID-19 patients (data were collected from 18 studies). They revealed the significance of ferritin in indicating a severe disease in 4992 patients and mortality risk in 2621 patients [30].

It remains unclear whether ferritin is a product of inflammation or a pathogenic mediator [31], and further research is needed. The values obtained for CRP and PCT are expected and agree with the results of numerous studies on inflammatory markers in COVID-19 patients.

Magnesium deficiency is discussed as a risk factor for severe COVID-19 infection [32], as it causes an increase in cytokine production and systemic inflammation. Additionally, hypermagnesemia increases the risk of death in the ICU [33] [34]. Hypermagnesemia could be caused by releasingmagnesium ions from its physiological store, the intracellular compartment, to the extracellular compartment [35]. Our study showed a significant increase in magnesium levels in the group of non-survivors during their stay in the ICU, similar to the observation in the Sharma et al. [35] study. Magnesium concentration is a parameter that should be carefully monitored in severe COVID-19 patients.

Some limitations of this study should be underlined, including its retrospective nature and the relatively small sample size, which may have impacted the statistical analysis.

## Conclusion

Our study showed that WBC, NEU, NLR, SII, D-dimer and bilirubin had significant potential as predictive biomarkers for poor outcomes in COVID-19 patients. Patients with values of NLR above 8 had a three times higher risk for lethal outcomes than those below 8. In addition, magnesium concentration significantly increased over time in the group of deceased patients while, at the same time, the haemoglobin level and PLT count decreased.

## Dodatak

### Conflict of interest statement

All the authors declare that they have no conflict of interest in this work.

### List of abbreviations

ALT, alanine aminotransferase;<br>AST, aspartate aminotransferase;<br>AUC, area under the curve;<br>CRP, C- reactive protein;<br>ICU, intensive care unit;<br>IL-6, Interleukin-6;<br>LDH, lactate dehydrogenase;<br>LYM, lymphocytes;<br>NEU, neutrophils;<br>NLR, neutrophile-to-lymphocyte ratio;<br>PLR, platelet-to-lymphocyte ratio;<br>PLT, platelets;<br>PCT, procalcitonin;<br>aPTT, activated partial thromboplastin time;<br>pCO2, partial pressureof carbon dioxide;<br>RI, reference interval;<br>SII, systemic immune-inflammation index;<br>WBC, leukocytes.
